# Factors Influencing Chloride Ion Diffusion in Reinforced Concrete Structures

**DOI:** 10.3390/ma17133296

**Published:** 2024-07-04

**Authors:** Qiulang Xu, Bin Liu, Lin Dai, Maogui Yao, Xijun Pang

**Affiliations:** 1 Guangxi Nanbin Highway Construction and Development Co., Ltd., Nanning 530400, China; 18877831029@163.com (Q.X.); 18269025556@163.com (L.D.); 18577668891@163.com (M.Y.); 2 Hualan Design & Consulting Group, Nanning 530011, China; liubin2014@hnu.edu.cn; 3 College of Civil Engineering and Architecture, Guangxi University, Nanning 530004, China

**Keywords:** chloride ions, diffusion coefficient, water-to-cement ratio, additive, anti-corrosion measurement

## Abstract

Reinforced concrete structures are prone to the corrosion of steel bars when exposed to chloride-rich environments, which can severely impact their durability. To address this issue, a comprehensive understanding of the factors influencing chloride ion diffusion in concrete is essential. This paper provides a summary of recent domestic and foreign research on chloride ion transport in concrete, focusing on six key factors: water–binder ratio, additive content, crack width, ambient temperature, relative humidity, and dry–wet cycles. The findings show that the diffusion coefficient of chloride ions in concrete increases with a higher water–binder ratio and decreases with increased additive content. Additionally, wider cracks result in a greater diffusion of chloride ions. The permeability resistance of concrete to chloride ions decreases with rising temperature and humidity, and dry–wet cycles further accelerate the diffusion of chloride ions. The article concludes by discussing various anti-corrosion measures, such as the use of corrosion inhibitors, surface coatings, and electrochemical treatments, to ensure the longevity of the structure. Finally, directions for future research are proposed.

## 1. Introduction

Reinforced concrete structures are extensively used in practical engineering applications, with their durability being a focal point of academic research [1,2]. One of the primary causes of reduced durability is the corrosion of steel reinforcement induced by chloride salts. Studies have shown that in high alkaline environments, a passivation film forms on the surface of the steel, maintaining a stable state [3,4]. However, when the structure is exposed to chloride environments for prolonged periods, chloride ions from the external environment diffuse from the concrete surface to the steel surface due to the concentration gradient. Once the chloride ion concentration at the steel surface exceeds a critical threshold, the passivation film transitions from a stable to an active state, leading to pitting corrosion of the steel surface [5,6,7]. The sources of chloride ions causing steel corrosion are twofold [8]: (1) chlorides mixed into the concrete and (2) chlorides from external environments, such as marine settings or de-icing salts. The former can be optimized by controlling the concrete mix materials, while the latter is influenced by uncontrollable factors. Chloride ions in concrete exist in three forms: free, physically adsorbed, and chemically bound, with free chloride being the main cause of steel corrosion [9]. Corrosion reduces the effective cross-sectional area of the steel, weakens the bond between steel and concrete, and causes the volume of corrosion products at the steel/concrete interface to expand by 2–4 times, leading to cracking and spalling of the protective layer, ultimately resulting in structural failure [10].

In recent years, extensive research has been conducted on chloride ion transport models, corrosion mechanisms, and their influencing factors [10,11,12]. Current chloride ion transport models are derived from Fick’s laws, with modifications based on experiments to account for factors such as the water–cement ratio, type and amount of additives, chloride binding capacity, and environmental conditions. However, due to the complexity of the factors influencing chloride ion diffusion and the intricate nature of the transport and corrosion processes, existing models still cannot accurately reflect the diffusion characteristics of chloride ions. Additionally, corrosion induced by chloride salts has led to significant structural damage, greatly increasing maintenance costs. To ensure structural durability, considerable attention has been paid to researching and applying measures to prevent steel corrosion. For critical structures in harsh environments, implementing appropriate additional measures can further mitigate chloride ion diffusion [13,14,15,16,17].

This paper summarizes the current state of research on chloride ion diffusion in concrete and measures to prevent steel corrosion. It reviews chloride ion diffusion models, analyzes key factors affecting chloride ion diffusion, and compares several commonly used additional measures to slow down steel corrosion, providing valuable insights for practical engineering applications.

## 2. Models for Chloride Ion Diffusion

The transfer of chloride ions in cementitious materials occurs through a combination of diffusion, convection, and electromigration. However, the dominant mode of transfer is dependent on the internal structure and external conditions of the concrete, and is influenced by the porosity and heterogeneity of the material. In cases where the pores are saturated with solution, diffusion becomes the dominant mode of chloride ion transfer in concrete. This diffusion is often non-steady-state and can be estimated using Fick’s second law, which states that the apparent diffusion coefficient of chloride ion under non-steady-state conditions can be estimated in one dimension:

(1) $$-J\left(x\right)=\frac{\partial C_{\left(x,t\right)}}{\partial t}=D\frac{dC_{\left(x,t\right)}^{2}}{dx^{2}}\;$$

Assume that the chloride diffusion coefficient *D* is constant, the chloride diffuses in a semi-infinite space, and the ion content at the surface of the concrete is invariable. The analytical solution for Equation (1) is

(2) $$C_{\left(x,t\right)}=C_{s}\left[1-erf\left(\frac{x}{2\sqrt{D\cdot t}}\right)\right]$$

This idealized approach does not take into account the physical and chemical interactions of chloride ions with the cementitious matrix, which can act as a sink or source for ion diffusion. This interaction results in a time-varying concentration of chloride ions at the surface of concrete and eventual equilibrium with the surrounding environment. Furthermore, the diffusion coefficient can vary depending on the heterogeneity of the concrete. To account for these complexities, Fick’s second law has been refined in various sophisticated forms, as highlighted in Table 1.

## 3. Factors Influencing Chloride Ion Diffusion

### 3.1. Water-to-Binder Ratio

**Figure 1:**
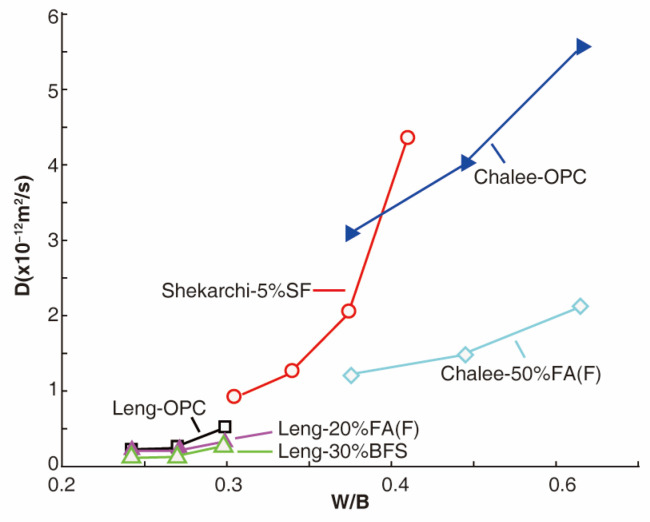
Chloride diffusion coefficient increases with the W/B ratio [26,28,34]. Note: OPC = ordinary Portland cement concrete, FA = fly ash, BFS = blast furnace slag, and SF = silica fume; X% represents X% of the cement weight replaced by X% of the additives added.

The water-to-binder (W/B) ratio, which is defined as the ratio of water to cementitious materials (such as cement and additives), is a crucial factor that affects the durability of concrete. Lowering the W/B ratio can effectively improve the pore structure and resistance to chloride ion penetration. The hydration of cementitious materials can densify the pore structure of concrete, reducing the size of pores and the diffusion of chloride ions [26,27]. It can be seen from Figure 1 that as the W/B ratio decreases, the chloride diffusion coefficient of the hardened concrete also decreases, as demonstrated in previous studies [28,29,30]. In addition to the W/B ratio, the presence of specific chemical compounds in the cement also affects the resistance to chloride ion penetration. For instance, the reaction of C_3_A and C_4_AF with chloride ions leads to the formation of Friedel salts, which can reduce the diffusion of chloride ions [26,31,32]. A study found that when the C_3_A content increased from 2.43% to 14%, the chloride ion threshold required for steel bar corrosion increased by 2.85 times [33]. In general, a low W/B ratio can effectively improve the pore structure of concrete, enhancing the chloride ion binding capacity, thereby effectively inhibiting chloride ion diffusion. In addition, using high pozzolan replacements in concrete, such as fine blast furnace slag or silica fume, can also lower the diffusion of chloride ions in concrete compared to ordinary Portland cement [26,34]. Thus, a combination of a low W/B ratio and the use of high pozzolan replacements can further improve the resistance of concrete to chloride ion penetration.

### 3.2. Additives

The resistance of concrete to chloride ion penetration can be improved by incorporating additives, such as fly ash, blast furnace slag, silica fume, and metakaolin. The basic parameters of each additives are shown in Table 2. The effectiveness of additives in resisting chloride penetration depends on specific surface area and active ingredient content [35,36,37,38]. The small particles and high specific surface area of additives act as micro-fillers between cement and aggregate particles, leading to a decrease in the total porosity and average pore size of cement slurry and hardened concrete [39,40,41,42]. Active additives can further improve the pore structure by reacting with hydrated products, such as pozzolanic materials that can react with portlandite to form calcium silicate hydrate, which has the ability to absorb chloride ions and densify the pore structure [34].

**Figure 2:**
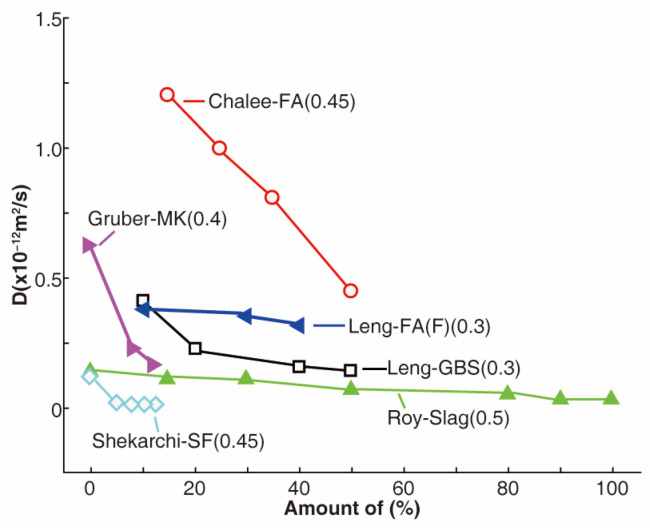
Chloride diffusion coefficient of concrete decreases with additives [26,34,43,44,45]. Note: Data in the brackets stand for W/B ratio; FA = fly ash, GBS = granulated blast furnace slag, SF = silica fume, and MK = high-reactivity metakaolin.

Studies have demonstrated that additives can greatly improve the resistance of concrete to chloride ion penetration, as shown in Figure 2 [26,34,43,44,45]. A small addition of additives, such as fly ash, can reduce the chloride diffusion coefficient by 15–50% [22]. Meanwhile, replacing 5–10% of cement with silica fume reduces the chloride diffusion coefficient by 50% [46,47]. However, a further increase in silica fume content beyond 7.5% has a limited effect on the chloride diffusion coefficient [34]. The addition of 8–12% metakaolin reduces the chloride diffusion coefficient by 50–60% [44]. In addition, binary or ternary mixtures of fly ash, silica fume, and slag in concrete have better resistance to chloride ion penetration than ordinary concrete and effectively reduce corrosion rates [37,48,49].

Generally, additives exhibit the capability to mitigate chloride ion diffusion and enhance their resistance to diffusion. However, excessive additives content can constrain their micro-filler effect and interaction with volcanic ash. This limitation may arise from an imbalance caused by high additives content, resulting in an insufficient Ca(OH)_2_ content in the hydration products of cement and thereby impeding the full utilization of volcanic ash reactions. Consequently, a substantial portion of unhydrated additive particles within concrete is susceptible to transport through internal and external convections, leading to heightened porosity within the concrete matrix. Consequently, as additives content increases, a phenomenon emerges where the resistance to chloride ion penetration in concrete tends to decrease rather than increase.

### 3.3. Cracks

**Figure 3:**
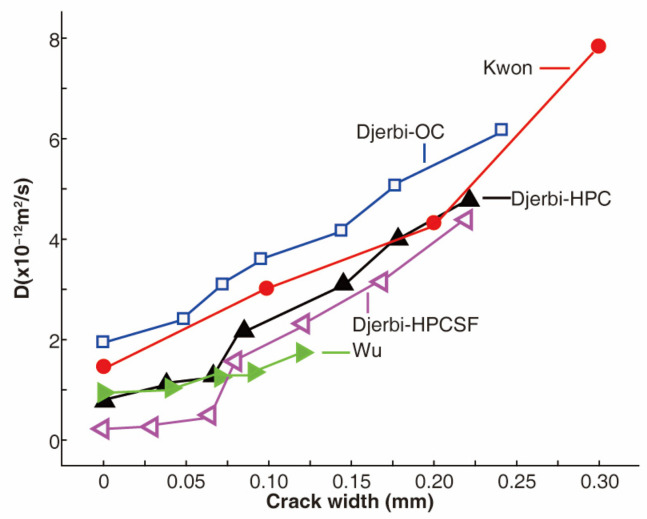
Chloride diffusion coefficient increase linearly with the crack width [52,53,54]. Note: OC = ordinary concrete, HPC = high-performance concrete, and HPCSF = high-performance concrete containing silica fume.

Cracks in concrete are unfortunate, but a widespread issue that serves to enlarge the pores of concrete, providing an opportunity for moisture and corrosive substances to penetrate the material [50,51]. The relationship between the crack width and the diffusion of chloride ions in concrete has been extensively studied and confirmed [52,53,54,55,56,57,58]. Kwon and colleagues [52] observed that the diffusion coefficient of chloride ions in concrete increased linearly with the crack width, as determined by analyzing the chloride diffusion of a pier that had been in use for 8 and 11 years (Figure 3). The same trend has been reported by other researchers who performed steady-state migration tests on pre-cracked concrete [53]. When cracks are present, the diffusion of chloride ions can increase by a factor of 2.5 to 7.9 times compared to uncracked concrete [59]. This acceleration of chloride ion diffusion results in the quicker corrosion of the reinforcing steel bars, and the byproduct of this corrosion, Fe(OH)_3_, exacerbates the issue by expanding the cracks and further facilitating the ingress of chloride ions and the rate of corrosion.

The impact of crack width on the diffusion of chloride ions in concrete is negligible when the crack width is below the critical crack width. When the crack width surpasses the critical crack width, however, the chloride ion diffusion rate spikes [60,61]. This critical crack width can vary. Research conducted through unsteady-state migration tests found that a crack width between 0.1 mm and 0.4 mm had a significant effect on the chloride diffusion coefficient of concrete [62]. The effect was small for crack widths less than 0.1 mm and approached a constant when the width was greater than 0.4 mm. Another study found that the critical value of the crack width for a specific concrete was 0.09 mm, where the chloride diffusion coefficient increased gradually with crack width and then steeply [54]. The critical crack width for steel fiber-reinforced concrete was determined to be 0.2 mm [63], above which the diffusion of chloride ions was significantly impacted by the crack. Other factors, such as crack density and curvature, may contribute to the varying critical crack widths [64].

### 3.4. Environmental Temperature

The hydration time of concrete can have a significant impact on its microscopic pore structure, which can vary based on the temperature of the environment. According to Care [65], heat-treating cement pastes at temperatures of 45 °C, 80 °C, and 105 °C resulted in an increase in the total porosity of the cement slurry, accompanied by the enlargement of pores. This finding was also echoed in studies that thermally treated concrete at high temperatures [66,67,68,69]. This is because high temperature conditions can lead to a “roughening effect”, resulting in concrete and cement with larger porosities and more diffuse paths for the diffusion of chloride ions.

The binding capacity of chloride ions in the concrete matrix is significantly impacted by temperature. On the one hand, as the temperature increases, calcium silicate hydrate decomposes, physically releasing the combined chloride [66]. On the other hand, the elevated temperature accelerates the hydration rate, thereby enhancing the chemical combination of chloride in the hydrated paste [70]. Research has shown that the combination capacity of chloride ions in the hydrated paste increases linearly with temperature within the range of 5 °C to 22 °C. In addition, temperature has a profound influence on the chloride ion threshold, above which concrete rebar begins to corrode. Hussain et al. conducted experiments on concrete rebar corrosion and found that the chloride ion threshold decreases by at least 80% when the concrete is exposed to temperatures ranging from 20 °C to 70 °C [33]. In other words, at higher temperatures, rebar in concrete begins to corrode at a lower chloride content. At higher temperatures, the [Cl^−^/OH^−^] ratio in the concrete pores increases as the chloride that was previously combined with the concrete paste is released, thereby decreasing the pore pH.

**Figure 4:**
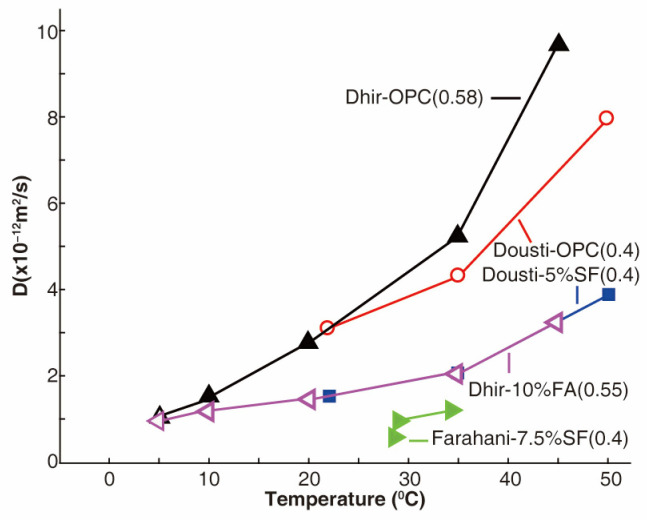
Coefficient of chloride ion diffusion increases with temperature [70,74,75]. Note: Data in the brackets stand for the water-to-cement ratio.

Temperature also changes the diffusion ratio of chloride ions in concrete. At higher temperatures, chloride ions become more active and exhibit a greater diffusion rate [71,72,73]. Although the addition of pozzolan additives can densify the concrete matrix and reduce diffusion, the influence of temperature on chloride ion diffusion is still notable [70,74,75], as indicated in Figure 4. Especially when the temperature exceeds 35 °C, the diffusion coefficient increases significantly. The main reasons for this phenomenon include [33,66,70,74,75]: First, at higher temperatures, the moisture within the concrete can evaporate more readily, creating micro-channels and reducing the internal resistance to ion movement. This increases the effective porosity and connectivity of the pore structure, facilitating faster ion diffusion. Second, elevated temperatures can alter the microstructure of concrete, such as causing thermal expansion or phase transformations in the cement matrix, which can reduce barriers to ion movement. Third, higher temperatures reduce the viscosity of the pore solution in concrete, making it easier for chloride ions to move through the pore solution. Moreover, increased temperature can accelerate chemical reactions between chloride ions and the cementitious materials, potentially changing the binding capacity and releasing more free chloride ions available for diffusion. Dousti found that high environmental temperatures result in a higher chloride ion content in concrete containing silica fume, with the penetration depth and diffusion coefficient of chloride ions increasing linearly with temperature. Similar findings have been reported by Farahani [75]. Dhir et al. [74] discovered that when 20% fly ash was used to replace cement, the diffusion coefficient of fly ash containing concrete decreased as the temperature increased, due to the reduction in the diffusion coefficient caused by the addition of fly ash exceeding the increase in the diffusion coefficient caused by a temperature rise. As such, it is crucial to choose a chloride diffusion test that reflects the exposure environment of the concrete structure, as failure to do so may result in an overestimation or underestimation of the diffusion coefficient of the concrete.

### 3.5. Relative Humidity

**Figure 5:**
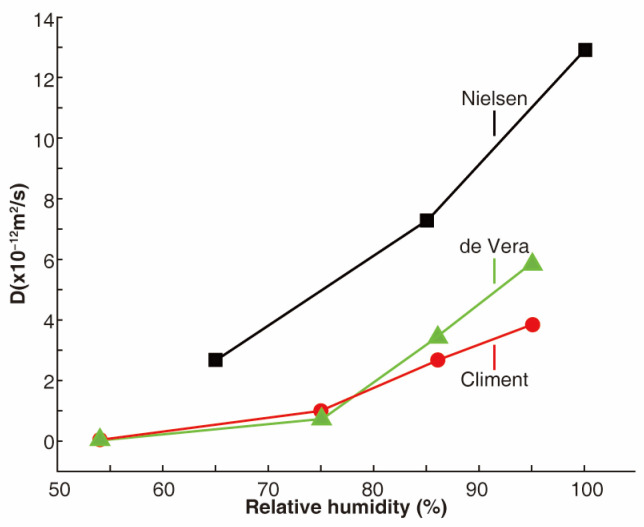
Chloride diffusion coefficient increases with the relative humidity of the concrete [79,80,81].

The relative humidity (typically referring to the humidity inside the concrete) has been shown to significantly impact the diffusion of chloride ions in concrete, due to its role as a carrier in the concrete’s porous structure [76,77,78]. Oh et al. [22] investigated the effect of relative humidity on chloride ion penetration in concrete by measuring the chloride profile of concrete specimens. They found that, as the relative humidity of the concrete increases, so does the accumulation of chloride ions on its surface. A compilation of the chloride diffusion coefficients for concrete samples, as reported in multiple studies, is presented in Figure 5. While the *D* values from the samples vary, it is clear that the chloride diffusion coefficient of concrete increases with rising relative humidity from 54% to 95% or even under vacuum saturation conditions [79,80,81]. The increase is nonlinear, with the diffusion of chloride ions in saturated pores being more effective than in partially saturated concrete, where the loss of pore water connectivity hinders the diffusion of chloride ions. At relative humidities lower than 70%, the chloride diffusion is relatively less affected. However, at higher humidities, the chloride diffusion is significantly impacted by the relative humidity.

### 3.6. Wet and Dry Cycle

Concrete, when exposed to the typical severe environmental conditions in marine and de-icing salt environments, particularly splash zones, is subjected to cycles of wetting and drying. These cycles have a significant impact on the internal pore structure of concrete, as has been demonstrated by previous research [82,83,84,85]. The cycles increase the porosity, critical pore size, and open porosity of the cement paste, providing more pathways for chloride ion transport and leading to deeper chloride ion penetration with increased concentration. However, the internal moisture distribution of concrete is only minimally affected by the cycles of wetting and drying. Research has shown that the changes in internal moisture under these conditions only occur within a specific depth range, referred to as the impact depth, where the relative internal humidity of the concrete undergoes cyclic changes [82].

The concrete experiences a flow between saturation and unsaturation under the alternation of cycles of wetting and drying. When the environment is moist, the internal humidity of the concrete rapidly increases to saturation within a short time, and chloride ions diffuse into the concrete along with the water. When the environment is dry, the internal water evaporates, causing the relative humidity to gradually decrease and the chloride ions to crystallize within the impact depth. This results in surface chloride ions being transported and accumulated inward under the influence of capillary suction [24,86]. As a result of the concrete structure being subjected to cycles of wetting and drying for an extended period, the diffusion of chloride ions is accelerated and the corrosion process is hastened. Studies have shown that the chloride ion content of samples under these cycles is significantly higher than that of samples under complete immersion conditions, and the chloride ion permeability increases over time [82,86]. Previous research [86] has also shown that the cycles of wetting and drying have a significant impact on the chloride ion diffusion and distribution in fly ash and slag concrete, and the chloride ion resistance of the concrete decreases over time.

## 4. Anti-Corrosion Measurements

Basic measurements, such as a low water–cement ratio, the addition of additives, increasing the thickness of the protective layer appropriately, and limiting the width of cracks, can effectively slow the corrosion of reinforcement, extending the service life of reinforced concrete structures. When the structure is in a harsh environment, in addition to the basic measures, additional measures are also needed to control the corrosion of reinforcement and extend the service life of the structure. These additional measures may include the addition of corrosion inhibitors, surface treatment, or electrochemical treatment (as seen in Table 3).

### 4.1. Corrosion Inhibitors

Corrosion inhibitors, also known as inhibitors, are one of the most commonly used effective methods for controlling the corrosion of steel reinforcements, particularly in the production of high-performance concrete. From a chemical-composition perspective, corrosion inhibitors can be divided into two categories: inorganic and organic. Inorganic inhibitors mainly include nitrite, chromate, molybdate, phosphate, and others [98]. Among these, nitrite is one of the earliest and commercially available anodic inhibitors. Anodic inhibitors react with metal ions at the anode to form a protective film on the metal surface, thus inhibiting the corrosion reaction, such as nitrite and chromate; while cathodic inhibitors, such as polyphosphates, reduce the cathodic reaction rate by forming sparingly soluble salts through reactions with liquid-phase ions at the cathode. Cathodic inhibitors only play a role when the doping amount is large, and their effect is usually not as effective as that of anodic inhibitors. Studies [87,88] have shown that calcium nitrite has a good corrosion inhibiting effect, can significantly increase the threshold level of chloride ions, reduce the corrosion rate of steel reinforcements, and extend the corrosion initiation time. However, nitrite and chromate have some adverse effects on human safety and environmental protection, and have been banned or restricted in countries such as Europe and America.

Researchers have discovered that organic compounds can effectively control steel reinforcement corrosion. Currently, commonly used organic corrosion inhibitors include alkyl amine, amine and amino acid, imidazoline, fatty acid ester, among others. Nitrogen, oxygen, sulfur atoms, and multiple bonds in organic molecules contribute to the corrosion inhibitor adhering to the metal surface, forming an organic layer, thereby inhibiting cathode–anode reactions [89]. Organic corrosion inhibitors can be incorporated into concrete mixtures, and some can be applied to the surface of hardened concrete, playing a protective role by migrating to the steel reinforcement’s surface. For example, studies [99,100] have proposed the use of alcohol amine migratory corrosion inhibitors (MCIs) for the surface of hardened concrete, where the active molecules migrate to the steel reinforcement’s surface to form a protective film, which can increase the concrete’s resistivity and significantly reduce the corrosion rate of the steel reinforcement.

Due to the harmfulness, slow biodegradability, and environmental restrictions of most synthetic inhibitors, the development of natural, non-toxic, harmless, and biodegradable green inhibitors has become the focus of research [101]. Satapathy et al. [90] found that compounds extracted from natural plants can be used as inhibitors, such as leaves and seeds. Plant extracts can serve as natural raw materials for green inhibitors, with simple extraction methods and a low cost, and are biodegradable in nature. Thus, green inhibitors have been widely developed and applied [102,103,104].

### 4.2. Concrete Coating

Surface coatings applied to concrete are a commonly employed method to prevent corrosion by blocking or slowing down the diffusion of chloride ions. Polymer coatings, such as epoxies, acrylics, and polyurethanes, are often used due to their hydrophobic properties and their ability to form a protective film on the concrete surface. This film acts as a physical barrier, effectively reducing the concentration and penetration rate of chloride ions on the concrete. Research conducted by Almusallam et al. [92] demonstrated that polymer coatings, including polyurethane, acrylic, and epoxy resins, exhibit an anti-chloride ion diffusion performance that is approximately ten-times greater than that of uncoated concrete. Furthermore, their results show that oxygen and polyurethane coatings exhibit a better performance than acrylic coatings. In addition, Jones et al. [71] found that the addition of silane to the coating serves as a corrosion inhibitor, significantly reducing the corrosion rate of steel bars. However, despite their benefits, the protective effect of organic coatings can be limited by their degradation over time due to exposure to ultraviolet radiation and environmental factors, such as waves. To address this issue, the integration of nanomaterials, such as clay minerals and carbon nanotubes, into polymer coatings has been found to enhance their protective effect on concrete. These nanomaterials are capable of filling the pores of the coating, increasing the diffusion path of chloride ions and reducing their concentration [93]. Furthermore, they improve the physical, mechanical, and thermal properties of polymers, and positively impact their biodegradation and stability [94]. The addition of these nanomaterials can thus extend the effective lifespan of the coatings and improve their overall performance in protecting concrete structures from corrosion.

In addition to utilizing corrosion inhibitors, surface treatments of concrete structures often involve the application of polymer-modified cement-based coatings. These coatings, which are comprised of polymer, cement, and fine aggregate, are a commonly used option for protecting concrete surfaces. In comparison to traditional polymer coatings, polymer-modified cement-based coatings exhibit increased resistance to ultraviolet (UV) radiation and improved toughness of the cement paste. This is achieved through the formation of a network structure within the hardened cement, which helps to reduce the presence of surface microcracks. This reduction in surface cracks enhances the suitability of polymer-modified cement-based coatings for crack repair [95]. Furthermore, the small porosity of these coatings effectively restricts the diffusion of chloride ions, reducing the likelihood of steel corrosion. Another coating option that has garnered interest is geopolymers, which have been shown to possess exceptional corrosion resistance in seawater environments and have been explored as a protective coating solution for marine concrete [105,106].

### 4.3. Electrochemical Treatment

The use of electrochemical treatment, which encompasses cathodic protection and electrochemical dechlorination, has been explored as a means to mitigate steel corrosion in concrete structures. The fundamental principle behind these methods is the application of an external electrical current to alter the local environment surrounding the steel bar. By making the steel bar cathodic, the process removes chloride ions and generates hydroxide ions on its surface, which raises the pH value and maintains the passivating performance of the steel, thus effectively inhibiting corrosion [107].

The application of cathodic protection involves the imposition of an external cathodic current on the surface of the steel bar, thus reducing its potential to a certain level by shifting it negatively. The cathodic reduction reaction that occurs during the application generates hydroxide ions and increases the pH value, inhibiting the corrosion of the steel bar [96]. There are two general approaches to cathodic protection: one involves a low current density of 0.5 to 2 mA/m^2^ to raise the critical chloride ion content required for pitting corrosion, while the other entails a current density as high as 15 or 20 mA/m^2^ to prevent corrosion from chloride salts and encourage their departure from the steel–concrete interface [108]. To be effective, it is essential to set an appropriate current density based on the existing chloride content to prevent its intrusion and impede its diffusion. The long-term application of cathodic protection is typically required for optimal inhibition of steel bar corrosion, but intermittent cathodic protection can be used in tidal areas and similar environments to reduce maintenance costs over the long term.

The electrochemical dechlorination process offers advantages over cathodic protection in terms of cost and time savings, while being effective in inhibiting steel corrosion. Similar to cathodic protection, the working principle of electrochemical dechlorination involves applying an external current to the steel bar. However, electrochemical dechlorination utilizes higher current densities, ranging from 0.5 A/m^2^ to 5 A/m^2^, to accelerate chloride ion migration [109,110,111]. The cathodic reaction generates hydroxide ions, increasing the pH value and promoting the re-passivation of the steel bar [112]. While electrochemical dechlorination can improve the internal structure of concrete, there are potential drawbacks to consider. Increased alkali content in the concrete pore solution can result in an alkali–aggregate reaction, causing concrete expansion and cracking, and hydrogen generated at the cathode may penetrate steel bars and cause hydrogen embrittlement [97]. Studies have reported conflicting results on the impact of electrochemical treatment. For instance, Polder [113] found that, after 39 days of treatment with a current density of 1 A/m^2^ or 4 A/m^2^, the chloride ion content on the surface of the steel bar was reduced by 70% to 90%, and about 40% to 70% of the initial chloride ions were removed from the concrete. However, Almeida Souza et al. [114] found that higher water–cement ratio concrete had a higher removal efficiency. Further research is required to fully understand the impact of electrochemical treatment.

## 5. Research Outlook

Reinforced concrete structures are frequently used in environments that contain chlorides, and the corrosion of steel bars as a result of exposure to chloride salts has been a persistent challenge. While the diffusion of chloride ions in concrete has been extensively studied, there is a need for further research on the chloride ion diffusion process in concrete, with a particular focus on the following four aspects.

### 5.1. Theoretical Models

The current theory of chloride ion diffusion in concrete is derived from Fick’s second law of diffusion, but it has been subject to various modifications, and the revised model still struggles to accurately reflect the diffusion of chloride ions in real-world conditions. This is due to the presence of a number of empirical formula parameters and the difficulty of accounting for the influence of various external factors. The influence of important factors, such as the combination of chloride ions and the multidimensional intrusion of chloride ions, must be taken into account in the model, as well as the changes in *D* over time and space.

### 5.2. Long-Term Testing

A significant limitation of existing research on chloride ion diffusion is that much of it is based on indoor experiments or numerical simulations, which typically provide data results over a much shorter time period than the service life of concrete structures. Consequently, there is a need for long-term durability tests to better understand concrete’s resistance to chloride ion penetration, and to guide the design of real-world engineering schemes. Particularly for the long-term management of nuclear waste using concrete as a container, the issue of corrosion in this process is especially noteworthy [115]. The long-term monitoring of parameters related to structural durability enables the predictive maintenance of structures [116]. To ensure that the results of indoor tests can be effectively applied in practice, it will be important to link and unify these results with long-term test results obtained under actual working conditions.

### 5.3. Multi-Factor Analysis

Most current research on chloride ion diffusion has focused on single-factor analysis, but it is crucial to consider the influence of multiple factors simultaneously, as the internal and external factors affecting concrete are often different. A multi-factor coupling effect analysis could provide a more effective reference for the analysis of chloride ion diffusion in real-world engineering applications. Additionally, a numerical simulation of the chloride ion diffusion process in concrete should be combined with different physical fields to better simulate the transport of chloride ions. Combining simulation results with experimental results would make it possible to effectively quantify the time-dependent effects of numerical simulations on chloride ion diffusion.

### 5.4. Material Innovations

There has been a significant amount of research on the mechanism and laws of the influence of various materials and anti-corrosion measures used in concrete, but there is still room for improvement. Future research could focus on broadening the range of additives and corrosion inhibitors, and developing new materials that can replace cement and improve the chloride ion penetration resistance and durability of concrete, while also providing environmental benefits, such as agricultural waste products, such as sugarcane slag, rice husk, and others [117,118,119]. By exploring these areas, it will be possible to develop more effective strategies for preventing corrosion in reinforced concrete structures in chloride-rich environments.

## 6. Conclusions

Reinforced concrete structures are integral components of modern infrastructure, enduring varying environmental conditions throughout their lifecycle. Among the several challenges faced by concrete, chloride ion diffusion leading to the corrosion of embedded steel reinforcement is a prominent concern. In this review, we delve into the intricate mechanisms underlying chloride ion diffusion within concrete and explore effective corrosion mitigation measures.

One of the key factors influencing chloride ion diffusion is the water-to-binder ratio (W/B), alongside the incorporation of additives. A lower W/B ratio coupled with a higher additives content has been observed to significantly enhance the internal pore structure of concrete, thereby augmenting chloride ion binding capacity and consequently restraining diffusion. It is noteworthy that, while an increase in the W/B ratio exponentially escalates chloride ion diffusion coefficients, augmenting the dosage of additives exhibits an exponential decrease. However, excessive additives dosage may inadvertently diminish the effectiveness of chloride ion diffusion inhibition. Additionally, concrete structures typically operate under normal service conditions with inherent cracking. When the width of these cracks surpasses a critical threshold, chloride ion diffusion coefficients linearly escalate with crack width, albeit with limited influence. Furthermore, environmental factors, notably moisture acting as a transport medium for chloride ions, exhibit a profound impact. Elevated environmental temperatures lead to increased porosity and the physical release of bound chlorides, consequently reducing concrete’s resistance to chloride ion penetration with rising temperatures and relative humidity. Under cyclic wet–dry exposure, chloride ions within concrete continuously crystallize within their effective depth, facilitating their internal transport and accumulation, thereby expediting corrosion processes. Mitigating chloride ion diffusion and steel reinforcement corrosion necessitates a multi-faceted approach encompassing concrete material optimization, corrosion inhibitors, surface treatments, and electrochemical methods. These supplementary measures collectively serve to prolong the structural service life.

In light of the rapid economic development, the proliferation of infrastructure projects underscores the imperative of continually optimizing concrete corrosion and protection systems. The burgeoning costs associated with infrastructure maintenance and repair mandate the refinement and enhancement of concrete structures’ corrosion resistance and protective frameworks, thereby ensuring their prolonged functionality and economic viability in the face of evolving environmental challenges.

## Figures and Tables

**Table 1:** The refined forms of Fick’s second law.

Year	Refined Term	Refined Formula	Note	Limitations	Reference
1994	Time-dependent ion content	$D_{c}=D_{i}t^{-m}$ $C_{\left(x,t\right)}=C_{s}\left[1-erf\left(\frac{x}{2\sqrt{\frac{D_{i}}{1-m}t^{\left(1-m\right)}}}\right)\right]$	The chloride ion diffusion coefficient per unit of time is denoted as *D_i_*, and *m* is a constant	*D_i_* is difficult to be measured. *m* is an empirical W/B-related constant No other factors considered	Mangat et al. [18]
1998	A linear function boundary condition	$C_{\left(x,t\right)}=kt\left\{\begin{array}{c} \left(1+\frac{x^{2}}{2D_{c}t}\right)erf\left(\frac{x}{2\sqrt{D_{c}t}}\right)\\ -\left(\frac{x}{\sqrt{\pi D_{c}t}}\right)e^{-\frac{x^{2}}{4D_{c}t}} \end{array}\right\}$	Boundary condition: *C_x_* = *kt*, *x* = 0, *t* > 0; Initial condition: *C_x_* = 0, *x* > 0, *t* =0	Only boundary conditions are constant	Amey et al. [19]
	Boundary condition is set as a power function	$C_{\left(x,t\right)}=k\sqrt{t}\left\{\begin{array}{c} e^{-\frac{x^{2}}{4D_{c}t}}-\\ \left[\frac{x\sqrt{\pi }}{2\sqrt{D_{c}t}}erf\left(\frac{x}{2\sqrt{D_{c}t}}\right)\right] \end{array}\right\}$	Boundary condition: $C_{x}=k\sqrt{t}$, *x* = 0, *t* > 0; Initial condition: *C_x_* = 0, *x* > 0, *t* = 0		
1999	*D* is deemed temperature- and time-dependent	$D_{c}=D_{28}{\left(\frac{t_{28}}{t}\right)}^{m}$	*D*_28_ represents the chloride ion diffusion coefficient at 28 days; *m* is a constant	*m* is a time-dependent empirical constant	Thomas et al. [20]
2000	Adsorption and desorption of chloride ions are counted	$C_{t}=C_{b}+\omega_{e}C_{f}$ ${D_{c}}^{*}=\frac{D_{c}}{1+\frac{1}{\omega_{e}}\frac{\partial C_{b}}{\partial C_{f}}}$	${D_{c}}^{*}$ represents the apparent diffusion coefficient; *D*_*c*_ represents the effective diffusion coefficient; *C*_*t*_ represents the total amount of chloride ions; *ω*_*e*_ represents the evaporable water content; *C*_*b*_ represents the amount of bound chloride ions; *C*_*f*_ represents the amount of free chloride ions; ∂*C*_*b*_/∂*C*_*f*_ represents the chloride ion binding capacity	Complicated	Martín-Pérez et al. [21]
2007	D is deemed as temperature-, time-, porosity-, and humidity-dependent	$D_{c}=D_{c}^{R}f_{T}(T)\cdot f_{t}(t)\cdot f_{h}(h)$	$D_{c}^{R}$ represents the chloride ion diffusion coefficient under a specific reference condition; *f*_*T*_(*T*) represents the temperature factor; *f*_*t*_(*t*) represents the time factor; *f*_*h*_(*h*) represents the pore relative humidity factor	Boundary conditions and chloride combination are not counted	Byung Hwan Oh et al. [22]
2012	Modeling of chloride ion diffusion in concrete based on cement paste	$\begin{array}{c} D_{eff}^{max}=D_{cem}\phi \\ D_{eff}^{min}=D_{cem}\phi \left(1+\phi \right)\left(3-\phi \right) \end{array}$	$D_{eff}^{max}\;$$\;\mathrm{and}\;D_{eff}^{min}\;$ represent the upper and lower bounds of the effective chloride ion diffusion coefficient in concrete, respectively; *ϕ* represents the volume fraction of the cement paste in concrete; *D_cem_* represents the chloride ion diffusion coefficient of the cement paste	The chloride ion diffusion coefficient of the cement paste must be known	Li et al. [23]
2016	Under loads and drying–wetting cycles	$C_{\left(x,t\right)}=C_{0}+0.00005\times \sqrt{t}\left\{e^{-x^{2}/\left(4\cdot f\left(p\right)D_{t}\right)}-\frac{x\sqrt{\pi }}{2\sqrt{{f\left(p\right)D}_{t}}}erf\left(\frac{x}{2\sqrt{{f\left(p\right)D}_{t}}}\right)\right\}$	$C_{s}=k\sqrt{t}$, and initial conditions and boundary conditions: ${C\left(x=0,t\right)=C}_{s},0<t<\infty ;$ ${C\left(x,t=0\right)=C}_{0},0<x<\infty$	—	Jin Wu et al. [24]
2020	Consider the influence of humidity, bonding effectiveness, and concrete crack size	$D_{Cl}=\left\{\begin{array}{c} D_{Cl}^{Sound}=D_{0}\cdot f\left(RH\right)\cdot f\left(C_{B}\right)\\ D_{Cl}^{Cr}=D_{Cl}^{Sound}\cdot f\left(w_{eff}\right) \end{array}\right.$	$D_{Cl}^{Sound}\;$$\;\mathrm{and}\;D_{Cl}^{Cr}$ represent the chloride ion diffusion coefficients of uncracked and cracked concrete, respectively; *D*_0_ represents the reference chloride ion diffusion coefficient, which is only related to the water–cement ratio of the concrete; *f*(*RH*) represents the humidity influence factor; *f*(*C*_*B*_) represents the binding influence factor; *f*(*w*_eff_) represents the effective width factor	—	Yu et al. [25]

**Table 2:** The basic parameters of additives [35].

Additive Types	Mean Size (μm)	Specific Surface Area (m^2^/g)	SiO_2_ (%)	Al_2_O_3_ (%)	Fe_2_O_3_ (%)	CaO (%)	Mg Ca(OH)_2_ Consumption per Gram
Fly ash (FA-F)	10~15	1~2	>50	20~30	<20	<5	850
Fly ash (FA-C)	10~15	1~2	>30	15~25	<10	20~30	500
Blast furnace slag (BFS)	—	—	32~38	8~16	<2	35~45	40
Silica (SF)	0.1~0.3	15~25	85~98	<2	<10	—	400
Metakaolin (MK)	1~2	~15	~55	35~45	<10	—	1000

Note: — indicates the lack of relevant data reports.

**Table 3:** Additional anti-corrosion measures.

Measure Type	Classification	Advantage	Shortcomings	Scope of Application	References
Corrosion inhibitor	Inorganic corrosion inhibitor	Easy to use, smaller dosage, remarkable effect, economical, and no loss of strength to concrete	Some corrosion inhibitors are poisonous and harmful and banned by many countries, such as nitrite	Incorporated into concrete or as a coating	[87,88]
	Organic corrosion inhibitor	Chemical bonds in organics facilitate adsorption on metal surfaces	Refractory and toxic		[89]
	Green corrosion inhibitor	Toxic, harmless, biodegradable, easy to obtain, and low cost	Corrosion inhibitors do not remove sources of corrosion in the field		[90]
Surface coating	Steel coating	Raise the steel bar corrosion threshold level and reduce the steel bar corrosion rate	Thickness of the galvanized coating is small, the reaction is fast, and the effective time is short; the steel coating may be insufficiently covered, debonded and degummed, and cannot be used for structural repair	Rebar surface coating	[91]
	Polymer coating	Small porosity, good hydrophobicity, and forms a physical barrier on the concrete surface	Limited UV resistance, susceptible to aging or damage by sea wave impact, shrinkage and deformation of the coating due to temperature changes, etc.	Concrete surface coating	[92]
	Polymer nanocomposite coating	Nanomaterials reduce coating porosity and improve polymer degradation and stability	—		[93,94]
	Polymer-modified cementitious coatings	Low porosity, good toughness, reduced surface microcracks, and good adhesion, flexibility, and UV resistance	—		[95]
Electrochemical treatment	Cathodic protection	Non-invasive and can increase the chloride ion threshold	Long application period and high cost	Widely used, but not suitable for prestressed structures	[96]
	Electrochemical dechlorination	Simple operation, high efficiency, low cost, time saving, no damage, and can improve the internal structure of concrete	There are side effects, such as the alkali–aggregate reaction and hydrogen embrittlement		[97]

Note: — indicates the lack of relevant research literature.

## Data Availability

The original contributions presented in the study are included in the article, further inquiries can be directed to the corresponding author.

## References

[1] Alexander M., Beushausen H. (2019). Durability, service life prediction, and modelling for reinforced concrete structures—Review and critique. Cem. Concr. Res..

[2] Qu F., Li W., Dong W., Tam V.W., Yu T. (2021). Durability deterioration of concrete under marine environment from material to structure: A critical review. J. Build. Eng..

[3] Pandey J.L., Banerjee M. (1998). Energy conservation with the use of solar selective coatings. Anti-Corros. Methods Mater..

[4] Papadakis V.G., Fardis M.N., Vayenas C.G. (1996). Physicochemical processes and mathematical modeling of concrete chlorination. Chem. Eng. Sci..

[5] Moreno M., Morris W., Alvarez M., Duffó G. (2004). Corrosion of reinforcing steel in simulated concrete pore solutions: Effect of carbonation and chloride content. Corros. Sci..

[6] Cruz R.V., Nishikata A., Tsuru T. (1998). Pitting corrosion mechanism of stainless steels under wet-dry exposure in chloride-containing environments. Corros. Sci..

[7] Ann K.Y., Song H.-W. (2007). Chloride threshold level for corrosion of steel in concrete. Corros. Sci..

[8] Arya C., Buenfeld N., Newman J. (1990). Factors influencing chloride-binding in concrete. Cem. Concr. Res..

[9] Hope B.B., Page J.A., Poland J.S. (1985). The determination of the chloride content of concrete. Cem. Concr. Res..

[10] Ahmad S. (2003). Reinforcement corrosion in concrete structures, its monitoring and service life prediction––A review. Cem. Concr. Compos..

[11] Yuan Q., Shi C., De Schutter G., Audenaert K., Deng D. (2009). Chloride binding of cement-based materials subjected to external chloride environment—A review. Constr. Build. Mater..

[12] Shi X., Xie N., Fortune K., Gong J. (2012). Durability of steel reinforced concrete in chloride environments: An overview. Constr. Build. Mater..

[13] Abdulrahman A., Ismail M., Hussain M.S. (2011). Corrosion inhibitors for steel reinforcement in concrete: A review. Sci. Res. Essays.

[14] Huang R., He S., Ou Y., Xiao L., Mei G. (2023). Experimental and numerical analysis of chloride ion transportation in concrete under bending load with reverse seepage. Appl. Ocean Res..

[15] Ding J., He S., Huang R., Xiao L., Mei G. (2023). Investigating the chloride ion resistance of cracked concrete through Reverse-seepage and Saturation based Action Anti-corrosion Tech (RS-AAT): Experimental and numerical analysis. Constr. Build. Mater..

[16] Raja P.B., Ismail M., Ghoreishiamiri S., Mirza J., Ismail M.C., Kakooei S., Rahim A.A. (2016). Reviews on corrosion inhibitors: A short view. Chem. Eng. Commun..

[17] Pan X., Shi Z., Shi C., Ling T.-C., Li N. (2017). A review on concrete surface treatment Part I: Types and mechanisms. Constr. Build. Mater..

[18] Mangat P., Molloy B. (1994). Prediction of long term chloride concentration in concrete. Mater. Struct..

[19] Amey S.L., Johnson D.A., Miltenberger M.A., Farzam H. (1998). Predicting the service life of concrete marine structures: An environmental methodology. Struct. J..

[20] Thomas M.D., Bamforth P.B. (1999). Modelling chloride diffusion in concrete: Effect of fly ash and slag. Cem. Concr. Res..

[21] Martın-Pérez B., Zibara H., Hooton R., Thomas M. (2000). A study of the effect of chloride binding on service life predictions. Cem. Concr. Res..

[22] Oh B.H., Jang S.Y. (2007). Effects of material and environmental parameters on chloride penetration profiles in concrete structures. Cem. Concr. Res..

[23] Li L.-Y., Xia J., Lin S.-S. (2012). A multi-phase model for predicting the effective diffusion coefficient of chlorides in concrete. Constr. Build. Mater..

[24] Wu J., Li H., Wang Z., Liu J. (2016). Transport model of chloride ions in concrete under loads and drying-wetting cycles. Constr. Build. Mater..

[25] Yu S., Jin H. (2020). Modeling of the corrosion-induced crack in concrete contained transverse crack subject to chloride ion penetration. Constr. Build. Mater..

[26] Leng F., Feng N., Lu X. (2000). An experimental study on the properties of resistance to diffusion of chloride ions of fly ash and blast furnace slag concrete. Cem. Concr. Res..

[27] Luo Y., Zhu Q., Chen D., Liu C., Pan S. (2022). Steel rebar corrosion and corrosion-induced cracking in reinforced foamed concrete. Front. Mater..

[28] Chalee W., Jaturapitakkul C., Chindaprasirt P. (2009). Predicting the chloride penetration of fly ash concrete in seawater. Mar. Struct..

[29] Liu Y., Wu T., Hu Y., Kou D. (2023). Chloride ion diffusion in saturated lightweight aggregate concrete: A numerical investigation. Structures.

[30] Liu R., Li J., Xiao H., Yao D., Yang W. (2024). Chloride ion diffusion performance of concrete and its influence on scour resistance. Structures.

[31] Hossain K., Lachemi M. (2004). Corrosion resistance and chloride diffusivity of volcanic ash blended cement mortar. Cem. Concr. Res..

[32] Arya C., Buenfeld N., Newman J. (1987). Assessment of simple methods of determining the free chloride ion content of cement paste. Cem. Concr. Res..

[33] Hussain S., Al-Musallam A., Al-Gahtani A. (1995). Factors affecting threshold chloride for reinforcement corrosion in concrete. Cem. Concr. Res..

[34] Shekarchi M., Rafiee A., Layssi H. (2009). Long-term chloride diffusion in silica fume concrete in harsh marine climates. Cem. Concr. Compos..

[35] Mindess S., Young F., Darwin D. (2003). Concrete 2nd editio. Tech. Doc..

[36] Li C.-z., Song X.-b., Jiang L. (2021). A time-dependent chloride diffusion model for predicting initial corrosion time of reinforced concrete with slag addition. Cem. Concr. Res..

[37] Tang H., Yang Y., Peng J., Liu P., Zhang J. (2022). Test and Mesoscopic Analysis of Chloride Ion Diffusion of High-Performance-Concrete with Fly Ash and Silica Fume. Coatings.

[38] Zhang Y., Wu S., Ma X., Fang L., Zhang J. (2022). Effects of additives on water permeability and chloride diffusivity of concrete under marine tidal environment. Constr. Build. Mater..

[39] Poon C.-S., Kou S., Lam L. (2006). Compressive strength, chloride diffusivity and pore structure of high performance metakaolin and silica fume concrete. Constr. Build. Mater..

[40] Boddy A., Hooton R., Gruber K. (2001). Long-term testing of the chloride-penetration resistance of concrete containing high-reactivity metakaolin. Cem. Concr. Res..

[41] Cheewaket T., Jaturapitakkul C., Chalee W. (2010). Long term performance of chloride binding capacity in fly ash concrete in a marine environment. Constr. Build. Mater..

[42] Jin H., Li Z., Zhang W., Liu J., Xie R., Tang L., Zhu J. (2022). Iodide and chloride ions diffusivity, pore characterization and microstructures of concrete incorporating ground granulated blast furnace slag. J. Mater. Res. Technol..

[43] Chalee W., Jaturapitakkul C. (2009). Effects of W/B ratios and fly ash finenesses on chloride diffusion coefficient of concrete in marine environment. Mater. Struct..

[44] Gruber K., Ramlochan T., Boddy A., Hooton R., Thomas M. (2001). Increasing concrete durability with high-reactivity metakaolin. Cem. Concr. Compos..

[45] Roy D.M., Jiang W., Silsbee M. (2000). Chloride diffusion in ordinary, blended, and alkali-activated cement pastes and its relation to other properties. Cem. Concr. Res..

[46] Sandberg P., Tang L., Andersen A. (1998). Recurrent studies of chloride ingress in uncracked marine concrete at various exposure times and elevations. Cem. Concr. Res..

[47] Toutanji H., McNeil S., Bayasi Z. (1998). Chloride permeability and impact resistance of polypropylene-fiber-reinforced silica fume concrete. Cem. Concr. Res..

[48] Hooton R., Titherington M. (2004). Chloride resistance of high-performance concretes subjected to accelerated curing. Cem. Concr. Res..

[49] Scott A., Alexander M. (2007). The influence of binder type, cracking and cover on corrosion rates of steel in chloride-contaminated concrete. Mag. Concr. Res..

[50] Aldea C.-M., Shah S.P., Karr A. (1999). Effect of cracking on water and chloride permeability of concrete. J. Mater. Civ. Eng..

[51] Wang K., Jansen D.C., Shah S.P., Karr A.F. (1997). Permeability study of cracked concrete. Cem. Concr. Res..

[52] Kwon S.J., Na U.J., Park S.S., Jung S.H. (2009). Service life prediction of concrete wharves with early-aged crack: Probabilistic approach for chloride diffusion. Struct. Saf..

[53] Djerbi A., Bonnet S., Khelidj A., Baroghel-Bouny V. (2008). Influence of traversing crack on chloride diffusion into concrete. Cem. Concr. Res..

[54] Wu J., Diao B., Ye Y., Zheng X. (2017). Chloride diffusivity and life prediction of cracked RC beams exposed to different wet-dry ratios and exposure duration. Adv. Mater. Sci. Eng..

[55] Wang X., Ba M., Yi B., Liu J. (2024). Experimental and numerical investigation on the effect of cracks on chloride diffusion and steel corrosion in concrete. J. Build. Eng..

[56] Liu Q.-f., Hu Z., Wang X.-e., Zhao H., Qian K., Li L.-j., Meng Z. (2022). Numerical study on cracking and its effect on chloride transport in concrete subjected to external load. Constr. Build. Mater..

[57] Zhao R., Wang M., Guan X. (2023). Exploring exact effects of various factors on chloride diffusion in cracked concrete: ABAQUS-based mesoscale simulations. Materials.

[58] Otieno M., Alexander M., Beushausen H.-D. (2010). Corrosion in cracked and uncracked concrete–influence of crack width, concrete quality and crack reopening. Mag. Concr. Res..

[59] Jacobsen S., Marchand J., Boisvert L. (1996). Effect of cracking and healing on chloride transport in OPC concrete. Cem. Concr. Res..

[60] Yoon I.S., Schlangen E., Rooij M.R.d., Van Breugel K. (2007). The effect of cracks on chloride penetration into concrete. Key Eng. Mater..

[61] Park S.-S., Kwon S.-J., Jung S.H. (2012). Analysis technique for chloride penetration in cracked concrete using equivalent diffusion and permeation. Constr. Build. Mater..

[62] Wang H.-L., Dai J.-G., Sun X.-Y., Zhang X.-L. (2016). Characteristics of concrete cracks and their influence on chloride penetration. Constr. Build. Mater..

[63] Mangat P., Gurusamy K. (1987). Chloride diffusion in steel fibre reinforced marine concrete. Cem. Concr. Res..

[64] Jang S.Y., Kim B.S., Oh B.H. (2011). Effect of crack width on chloride diffusion coefficients of concrete by steady-state migration tests. Cem. Concr. Res..

[65] Care S. (2008). Effect of temperature on porosity and on chloride diffusion in cement pastes. Constr. Build. Mater..

[66] Xu Y., Wong Y., Poon C.S., Anson M. (2001). Impact of high temperature on PFA concrete. Cem. Concr. Res..

[67] Chan Y., Luo X., Sun W. (2000). Compressive strength and pore structure of high-performance concrete after exposure to high temperature up to 800 °C. Cem. Concr. Res..

[68] Poon C.-S., Azhar S., Anson M., Wong Y.-L. (2001). Comparison of the strength and durability performance of normal-and high-strength pozzolanic concretes at elevated temperatures. Cem. Concr. Res..

[69] Poon C.-S., Azhar S., Anson M., Wong Y.-L. (2003). Performance of metakaolin concrete at elevated temperatures. Cem. Concr. Compos..

[70] Dousti A., Rashetnia R., Ahmadi B., Shekarchi M. (2013). Influence of exposure temperature on chloride diffusion in concretes incorporating silica fume or natural zeolite. Constr. Build. Mater..

[71] Jones M., Dhir R., Gill J. (1995). Concrete surface treatment: Effect of exposure temperature on chloride diffusion resistance. Cem. Concr. Res..

[72] Samson E., Marchand J. (2007). Modeling the effect of temperature on ionic transport in cementitious materials. Cem. Concr. Res..

[73] Jin L., Yu H., Wang Z., Wang Z., Fan T. (2022). Developing a model for chloride transport through concrete considering the key factors. Case Stud. Constr. Mater..

[74] Dhir R., Jones M., Elghaly A. (1993). PFA concrete: Exposure temperature effects on chloride diffusion. Cem. Concr. Res..

[75] Farahani A., Taghaddos H., Shekarchi M. (2015). Prediction of long-term chloride diffusion in silica fume concrete in a marine environment. Cem. Concr. Compos..

[76] Chen D., Guo W., Wu B., Shi J. (2023). Service life prediction and time-variant reliability of reinforced concrete structures in harsh marine environment considering multiple factors: A case study for Qingdao Bay Bridge. Eng. Fail. Anal..

[77] Zacchei E., Bastidas-Arteaga E. (2022). Multifactorial Chloride Ingress Model for Reinforced Concrete Structures Subjected to Unsaturated Conditions. Buildings.

[78] Chen D., Feng Y., Shen J., Sun G., Shi J. (2022). Experimental and simulation study on chloride diffusion in unsaturated concrete under the coupled effect of carbonation and loading. Structures.

[79] Nielsen E.P., Geiker M.R. (2003). Chloride diffusion in partially saturated cementitious material. Cem. Concr. Res..

[80] Climent M.A., de Vera G., López J.F., Viqueira E., Andrade C. (2002). A test method for measuring chloride diffusion coefficients through nonsaturated concrete: Part I. The instantaneous plane source diffusion case. Cem. Concr. Res..

[81] de Vera G., Climent M.A., Viqueira E., Antón C., Andrade C. (2007). A test method for measuring chloride diffusion coefficients through partially saturated concrete. Part II: The instantaneous plane source diffusion case with chloride binding consideration. Cem. Concr. Res..

[82] Chang H., Mu S., Xie D., Wang P. (2017). Influence of pore structure and moisture distribution on chloride “maximum phenomenon” in surface layer of specimens exposed to cyclic drying-wetting condition. Constr. Build. Mater..

[83] Ju X., Wu L., Lin C., Yang X., Yang C. (2021). Prediction of chloride concentration with elevation in concrete exposed to cyclic drying-wetting conditions in marine environments. Constr. Build. Mater..

[84] Jin H., Liu J., Zhong D., Tang L. (2023). Experimental study on chloride ion diffusion behavior and microstructure in concrete under alternating ambient humidity conditions. Constr. Build. Mater..

[85] Wang Y., Guo S., Yan B., Liu Z., Wang Y., Yuan C. (2022). Experimental and analytical investigation on chloride ions transport in concrete considering the effect of dry-exposure ratio under diurnal tidal environment. Constr. Build. Mater..

[86] Hong K., Hooton R. (1999). Effects of cyclic chloride exposure on penetration of concrete cover. Cem. Concr. Res..

[87] Ann K.-Y., Jung H., Kim H., Kim S., Moon H.Y. (2006). Effect of calcium nitrite-based corrosion inhibitor in preventing corrosion of embedded steel in concrete. Cem. Concr. Res..

[88] Berke N.S., Hicks M.C. (2004). Predicting long-term durability of steel reinforced concrete with calcium nitrite corrosion inhibitor. Cem. Concr. Compos..

[89] Ormellese M., Berra M., Bolzoni F., Pastore T. (2006). Corrosion inhibitors for chlorides induced corrosion in reinforced concrete structures. Cem. Concr. Res..

[90] Satapathy A., Gunasekaran G., Sahoo S., Amit K., Rodrigues P. (2009). Corrosion inhibition by Justicia gendarussa plant extract in hydrochloric acid solution. Corros. Sci..

[91] Bautista A., González J.A. (1996). Analysis of the protective efficiency of galvanizing against corrosion of reinforcements embedded in chloride contaminated concrete. Cem. Concr. Res..

[92] Almusallam A., Khan F., Dulaijan S., Al-Amoudi O. (2003). Effectiveness of surface coatings in improving concrete durability. Cem. Concr. Compos..

[93] Scarfato P., Di Maio L., Fariello M.L., Russo P., Incarnato L. (2012). Preparation and evaluation of polymer/clay nanocomposite surface treatments for concrete durability enhancement. Cem. Concr. Compos..

[94] Kumar A.P., Depan D., Tomer N.S., Singh R.P. (2009). Nanoscale particles for polymer degradation and stabilization—Trends and future perspectives. Prog. Polym. Sci..

[95] Diamanti M.V., Brenna A., Bolzoni F., Berra M., Pastore T., Ormellese M. (2013). Effect of polymer modified cementitious coatings on water and chloride permeability in concrete. Constr. Build. Mater..

[96] Glass G., Chadwick J. (1994). An investigation into the mechanisms of protection afforded by a cathodic current and the implications for advances in the field of cathodic protection. Corros. Sci..

[97] Huang T., Huang X., Wu P. (2014). Review of recent developments of electrochemical chloride extraction on reinforced concrete in civil engineering. Int. J. Electrochem. Sci..

[98] Abd El Haleem S., Abd El Wanees S., Abd El Aal E., Diab A. (2010). Environmental factors affecting the corrosion behavior of reinforcing steel II. Role of some anions in the initiation and inhibition of pitting corrosion of steel in Ca(OH)_2_ solutions. Corros. Sci..

[99] Malik A.U., Andijani I., Al-Moaili F., Ozair G. (2004). Studies on the performance of migratory corrosion inhibitors in protection of rebar concrete in Gulf seawater environment. Cem. Concr. Compos..

[100] Morris W., Vazquez M. (2002). A migrating corrosion inhibitor evaluated in concrete containing various contents of admixed chlorides. Cem. Concr. Res..

[101] Asipita S.A., Ismail M., Abd Majid M.Z., Majid Z.A., Abdullah C., Mirza J. (2014). Green Bambusa Arundinacea leaves extract as a sustainable corrosion inhibitor in steel reinforced concrete. J. Clean. Prod..

[102] Palanisamy S., Maheswaran G., Selvarani A.G., Kamal C., Venkatesh G. (2018). Ricinus communis—A green extract for the improvement of anti-corrosion and mechanical properties of reinforcing steel in concrete in chloride media. J. Build. Eng..

[103] Mourya P., Banerjee S., Singh M. (2014). Corrosion inhibition of mild steel in acidic solution by *Tagetes erecta* (Marigold flower) extract as a green inhibitor. Corros. Sci..

[104] Liu Y., Song Z., Wang W., Jiang L., Zhang Y., Guo M., Song F., Xu N. (2019). Effect of ginger extract as green inhibitor on chloride-induced corrosion of carbon steel in simulated concrete pore solutions. J. Clean. Prod..

[105] Zhang Z., Yao X., Zhu H. (2010). Potential application of geopolymers as protection coatings for marine concrete: II. Microstructure and anticorrosion mechanism. Appl. Clay Sci..

[106] Zhang Z., Yao X., Wang H. (2012). Potential application of geopolymers as protection coatings for marine concrete III. Field experiment. Appl. Clay Sci..

[107] Marcotte T., Hansson C., Hope B. (1999). The effect of the electrochemical chloride extraction treatment on steel-reinforced mortar Part I: Electrochemical measurements. Cem. Concr. Res..

[108] Carmona J., Garcés P., Climent M. (2015). Efficiency of a conductive cement-based anodic system for the application of cathodic protection, cathodic prevention and electrochemical chloride extraction to control corrosion in reinforced concrete structures. Corros. Sci..

[109] Page C., Yu S. (1995). Potential effects of electrochemical desalination of concrete on alkali–silica reaction. Mag. Concr. Res..

[110] Ihekwaba N., Hope B., Hansson C. (1996). Pull-out and bond degradation of steel rebars in ECE concrete. Cem. Concr. Res..

[111] Saraswathy V., Lee H.-S., Karthick S., Kwon S.-J. (2018). Extraction of chloride from chloride contaminated concrete through electrochemical method using different anodes. Constr. Build. Mater..

[112] Elsener B. (2008). Long-term durability of electrochemical chloride extraction. Mater. Corros..

[113] Polder R.B. (1996). Electrochemical chloride removal from concrete prisms containing chloride penetrated from sea water. Constr. Build. Mater..

[114] de Almeida Souza L.R., de Medeiros M.H.F., Pereira E., Capraro A.P.B. (2017). Electrochemical chloride extraction: Efficiency and impact on concrete containing 1% of NaCl. Constr. Build. Mater..

[115] Rahman R.O.A., Ojovan M.I. (2021). Sustainability of Life Cycle Management for Nuclear Cementation-Based Technologies.

[116] Rebolledo N., Torres J.E., Silva A., Sánchez J. (2024). Monitoring of Reinforced Concrete Corrosion: Active and Passive Bars Exposed to Climate. Appl. Sci..

[117] Chandra Paul S., Mbewe P.B., Kong S.Y., Šavija B. (2019). Agricultural solid waste as source of supplementary cementitious materials in developing countries. Materials.

[118] Qin Y., Pang X., Tan K., Bao T. (2021). Evaluation of pervious concrete performance with pulverized biochar as cement replacement. Cem. Concr. Compos..

[119] Tan K., Pang X., Qin Y., Wang J. (2020). Properties of cement mortar containing pulverized biochar pyrolyzed at different temperatures. Constr. Build. Mater..

